# Rheological, physical, and mechanical properties of chicken skin gelatin films incorporated with potato starch

**DOI:** 10.1038/s41538-019-0059-3

**Published:** 2019-12-04

**Authors:** Syazwani Aqilah Alias, Norizah Mhd Sarbon

**Affiliations:** Faculty of Fisheries and Food Science, Universiti Malaysia Terengganu, 21030 Kuala Nerus, Terengganu Malaysia

**Keywords:** Biomaterials - proteins, Characterization and analytical techniques

## Abstract

The aim of this study was to investigate the rheological, physical, and mechanical properties of chicken skin gelatin film forming solutions (FFSs) and films incorporated with potato starch. Chicken skin gelatin-based FFSs with various potato starch concentrations (0, 2, 4, 6, 8, and 10%, w/w) were prepared via casting technique. The dynamic viscoelastic properties of FFS were measured, and film characterization in terms of physical and mechanical properties was conducted. Potato starch incorporation with chicken skin gelatin-based FFS resulted in improvement of viscous behavior (*G*″ > *G*′). As potato starch concentration increased, the tensile strength, elongation at break, and elastic modulus values of chicken skin gelatin-based films also increased (*p* < 0.05). Additionally, increasing the concentration of potato starch caused incremental changes in water vapor permeability and melting temperatures (*T*_m_), but a reduction in water solubility (*p* < 0.05). In addition, the surface smoothness and internal structure of composite films improved via potato starch incorporation. The incorporation of potato starch was also found to provide good barrier properties against ultraviolet and visible light, but did not significantly influence the transparency values of composite films. Overall, chicken skin gelatin film with 6% potato starch concentration incorporation was the most promising composite film, since it was found to exhibit optimal performance in terms of physical properties.

## Introduction

Food packaging is a coordinated enclosure system employed in preparing food for transport, distribution, storage, retailing, and end-use. Manufacturers aim for optimal cost and ultimate consumer satisfaction.^1^ Packaging material is crucial to food product development, as it is responsible for preserving, protecting, and containing a food product from production until consumption by the end consumer.^2^ Conventional synthetic polymer packaging depends on easy availability and thus low cost of production. However, the environmental impacts caused by poor biodegradability have recently led to growing interest in the development of biodegradable-edible packaging materials.

An edible film can be defined as a thin layer of material that can be eaten, but which provides a barrier to mass transfer within the food itself or between the food and the environment.^3^ By utilizing natural commodities such as polysaccharides, proteins, lipids, and blended composites, this type of packaging has attracted attention due to its many advantages over synthetic materials. For example, some such materials offer good protection against the environment.^4^ This was confirmed by Suderman et al.,^5^ who found that certain proteins and polysaccharides were the primary polymers used in making biodegradable-edible films. Proteins, specifically gelatin components, are commonly used as major ingredients in edible film development due to their superior film forming properties as compared with other non-protein sources.^6^ Examples include chicken skin gelatin film^7^ and fish gelatin film.^8^ However, prior studies have also uncovered the disadvantages of edible coatings, especially in gelatin-based films. These disadvantages include high water vapor permeability (WVP), moisture content and films solubility as compared to lipid and polysaccharide-based films.^7^

Numerous protein sources have been utilized for gelatin production, including pig skin,^9^ Tuna fish skin,^10^ and bovine skin.^11^ In addition, chicken skin gelatin shows potential as an alternative source due to its superior characteristics. While chicken skin gelatin has a similar chemical composition to bovine gelatin, it offers better physicochemical properties against reported fish gelatin.^12^ Therefore, numerous studies have been performed to confirm the functionalities of gelatin-based composite films, including rice starch-gelatin films,^13^ tapioca starch-gelatin films,^14^ tapioca starch-gelatin films,^15^ and carboxymethyl cellulose-gelatin films.^16^ However, due to its highly hydrophilic nature, edible films composed of gelatin have been found to have limited resistance against moisture. Hence, improvements in internal crosslinking through the development of a composite blended film matrix can be made to achieve better stability and extensibility.

Starch is more widely used than other polymers due to its excellent performance, specifically in terms of mechanical strength and oxygen barrier properties.^17^ Starches from different sources have been studied in order to determine their functionality, especially in the food packaging context, such as potato starch,^18^ corn starch,^19^ sago starch,^20^ and rice starch.^21^ Potato starch has recently been highlighted by numerous researchers as a potentially excellent incorporation agent due to remarkable characteristics such as high amylose content and large granule size. It offers many advantages in terms of transparency, paste clarity, and extensibility.^22^ Hence, the use of potato starch in the development of an edible composite film may improve its overall performance.

Ideally, the performance of packaging film can be confirmed by its functional properties, specifically the physical and rheological properties of a film forming solution (FFS). Rheological properties indeed play an important role in the production of high-quality composite films. They must be considered in optimizing the processing design, since they influence the spreadability, thickness and uniformity of FFS and the finalized film’s performance.^23^ The physical properties of films are determined in terms of tensile strength (TS), elongation at break (EAB), and Young’s modulus (YM).^13^^,14^ Moreover, physical properties of films were include water solubility, WVP, light transmittance and film transparency, thermal properties, and also morphology of films. A study by Nur Hazirah et al.^24^ demonstrated greater TS in composite blends of protein and polysaccharides compared to proteins or polysaccharides in isolation, thus confirming a better orientation network between those two biopolymer chains.

Therefore, the objectives of this study are to determine the viscoelastic properties of a chicken skin gelatin FFS and characterize the mechanical and physical properties of chicken skin gelatin film as affected by varying concentrations of potato starch.

## Results

### Dynamic viscoelastic properties

The rheological properties of chicken skin gelatin-potato starch films forming solution were measured within the linear viscoelastic region (LVR) with small-amplitude oscillatory testing in order to verify gelatinization and structure of gels in terms of viscous and elastic properties. From both *G*′ and *G*″ moduli, all chicken skin gelatin-potato starch FFS showed a strain range of 0.1–10% indicating a linear viscoelastic domain. Critical strain at 1% was defined as the limit strain of the LVR to mark the end of the linear stress–strain relation.

**Fig. 1:**
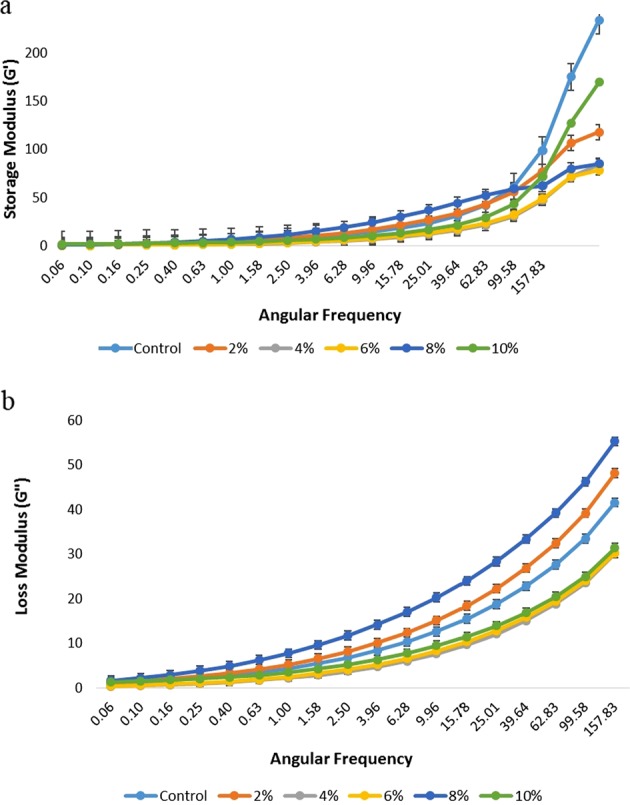
**a** Frequency sweep curved of the storage or elastic modulus (*G*′) of chicken skin gelatin-potato starch film forming solution at different formulation. **b** Frequency sweep curved of the loss or viscous modulus (*G*″) of chicken skin gelatin-potato starch film forming solution at different formulations

Figure 1a, b shows the storage or elastic modulus (EM) (*G*′) and loss or viscous modulus (*G*″) of chicken skin gelatin-potato starch FFS at different formulations. Across the angular frequency range from 10 to 100 rad/s, all the FFS showed an increase in *G*′ upon frequency increased. FFS *E* (8% potato starch) was found to obtain higher EM (*G*′) compared to other formulated FFS. This is due to the potato starch polymer chains with increased concentration participating in the formation of a film matrix network structure.^25^ This resulted in a higher value for *G*′ as potato starch concentration increased. This result was similar to that of a study conducted by Huang et al.,^26^ where the *G*′ value of pectin-fish scale gelatin gel increased as the angular frequency increased. Meanwhile, focusing on the similar gelatin sources which chicken skin gelatin characterized by Sarbon et al.,^12^ chicken skin gelatin showed positive growth as represented by *G*′ moduli as frequency increased, hence confirming the stable gel network formed.

All FFS showed the similar dynamic viscoelastic behavior with a typical liquid-like response across 1–100 rad/s angular frequency, in which the loss modulus was greater than storage modulus (*G*″ > *G*′). This indicates that this chicken skin gelatin-potato starch-based FFS dominantly demonstrates viscous behavior. However, both moduli still had similar linearly incremented trends against increasing angular frequency, which indicates that *G*″ was also frequency dependence. This explains the potato starch content’s crucial role in enhancing structural integrity, providing more interacting starch chains for the formation of FFS network structure.^25,27^

These findings are in agreement with those of Valencia et al.,^28^ who reported similar typical liquid-like response for the whole frequency range tested on nanocomposite-forming solutions with cassava starch and low laponite up to 5%, as well as by chitosan and chitosan-starch with polyphenol-rich aqueous extract from Murta leaves incorporation^29^ that also implies consistent behavior (*G*″ > *G*′). In addition, considering the type of starch used in this composite FFS development, which is potato starch, several affect might be induced by the physicochemical properties of this particular starch. Due to its high molecular weight, gelatinized potato starch is able to produce high-viscosity paste.^30^ The large size of the potato starch granules and also great amylose content (29.3%, w/w) compared to other common starches such as tapioca starch (17%) and rice starch (20%) influence its swelling properties, which will help prevent water retention, thus allowing good solubility properties.^22^ In short, chicken skin gelatin-potato starch FFS with varied potato starch concentration had a liquid state solution (*G*″ > *G*′) in which the *G*″ moduli increased considerably against *G*′ as angular frequency increasing.

### TS of edible composite film

The effects of varied potato starch concentration on TS are shown in Table 1. There was no significant difference between all film formulations except for formulation B. The highest peak (2.76 MPa) was exerted by composite blended film D (6% potato starch).

**Table 1:** Tensile strength, elongation at break, and Young’s elastic modulus for chicken skin gelatin-potato starch edible composite film

Sample	Tensile strength (MPa)	Elongation at break (%)	Young’s elastic modulus (MPa)
A	2.11 ± 1.12^a^	65.82 ± 1.17^a^	0.03 ± 0.01^a^
B	1.78 ± 0.15^b^	38.57 ± 5.66^c^	0.05 ± 0.01^a^
C	2.69 ± 0.11^a^	64.51 ± 0.96^a^	0.04 ± 0.00^a^
D	2.76 ± 0.36^a^	50.92 ± 4.61^b^	0.05 ± 0.00^a^
E	2.09 ± 0.20^a^	46.54 ± 2.60^b^	0.05 ± 0.00^a^
F	2.01 ± 0.29^a^	34.80 ± 4.30^c^	0.06 ± 0.02^a^

*A* with 0% of potato starch, *B* with 2% of potato starch, *C* with 4% of potato starch, *D* with 6% of potato starch, *E* with 8% of potato starch, *F* with 10% of potato starch

All data represent mean ± standard deviation; the different superscript letter (^a–b^) in the same column indicate significant difference (*p* *<* 0.05). Values are expressed as mean ± SD (*n* = 3)

Increasing potato starch concentration led to better enhancement in structural integrity within the composite film matrix.^30^ The compatible interlace between gelatin and macromolecular potato starch chains induced strengthen mechanical resistance, as a strong structural network formed in between anionic groups of polysaccharides and cationic groups of gelatin.^31^ Moreover, potato starch added into a composite film matrix is known to act as crosslinker, which helps in lowered molecular mobility throughout optimization entanglement chain.^13^ However, TS values decreased at 8 and 10% potato starch concentration. This is because the high levels of biopolymers added into blended films decreased the compactness of the films, resulting in reduced TS values. These findings were comparable against the results from chicken skin gelatin-based film incorporated with tapioca starch,^14^ as similar trends were observed for 5–15% tapioca starch formulated films with TS value ranging from 2.42 to 2.85 MPa. However, lower TS values (1.28–1.67 MPa) were reported by Al-Hassan and Norziah^20^ for gelatin-based film with 2–5% sago starch incorporation, as compared to this study (1.78–2.76 MPa). The high amylose content within potato starch granules was found to be attributed to the aggressive formation of more hydrogen bonding against chicken skin gelatin.^22^ The increment of potato starch content helps in formation of chicken skin gelatin-based composite film with better strength as implies by maximum TS possessed by film D with the formulation of 6% potato starch incorporation.

### EAB of edible composite film

The elongations at break of chicken skin gelatin films with varied concentration of potato starch incorporation are shown in Table 1. From the findings, films A and C were found to be significantly different to films B, D, E, and F, respectively, upon increasing potato starch incorporation, which varied from 0 to 10% (*p* < 0.05).

Generally, this decreasing EAB trend was attributed to the desired amount of potato starch producing an apparent plasticizing effect, promoting chain mobility. This was influenced by the existence of crosslinking reaction imparted by interlacement of gelatin and large macromolecular potato starch chains. These decreases in further EAB values may be attributed to the formation of intermolecular interactions between potato starch and gelatin molecules, thus contributing to the reduction of film flexibility which also affected the EAB values of films.^32^ This is further explained by the inability of amylose inherent by potato starch to fora continuous network within the composite film matrix.^33^ However, an inappropriate level of starch concentration may lead to an inability to maintain a continuous network, which restrains the starch functional role as a crosslinker agent with composite blended film matrix.

### Young’s EM of edible composite film

Table 1 shows the effect of potato starch concentration on Young’s EM of chicken skin gelatin-based composite film. There were no significant differences between all formulations with increased potato starch levels. Young’s EM is the fundamental measure of the film stiffness as higher stiffness of material closely associated with higher EM values.^34^ The increment of potato starch incorporated into chicken skin gelatin-based blended film lead to a thicker film with higher TS and lower EAB, and might be the reason behind it. Further incorporation would help in producing a superior structural integrity of film matrix via the enhancement of chain entanglement in between gelatin and starch polymer chains, indirectly attributed to denser and stronger films, hence confirming the stiffness parameter relations.^21^

The findings obtained in this study are in agreement with a study conducted by Christine and Sarbon,^14^ who reported an increment trend followed by decreasing EM values with a further increase of starch content. The highest EM value possessed by film C with 10% tapioca starch incorporation (3.43 MPa), while the lowest value assisted by film B with 5% tapioca starch incorporation. This may be due to the dominant phase of gelatin component within the interaction between polysaccharides and gelatin film matrix, thus implies the stronger properties and elevated the high film’s stiffness. Therefore, the EM of chicken skin gelatin-potato starch films was influenced by the addition of potato starch due to film D (4% potato starch) having the highest EM value among all film formulations.

### Light transmission and film transparency determination

Ultraviolet (UV) light transmission (200–280 nm), visible light (350–800 nm) and transparency values at 600 nm for six different formulated chicken skin gelatin-potato starch films are shown in Table 2. From the table, it can be observed that chicken skin gelatin-potato starch films successfully blocked the UV light transmission with favorable absorption, as no collected transmittance results were verified for all films. Next, visible light transmission (350–800 nm) was found to be influenced by addition of potato starch. Film F, with the highest concentration of potato starch (10%), had the lowest visible light transmission of formulated films at wavelengths of 350 nm, 400 nm, 700 nm, and 800 nm, respectively, excluding wavelengths 500 nm and 600 nm. However, the transparency value at 600 nm recorded for the films produced an insignificant decline from 1.08 to 0.94 nm with increased potato starch incorporation across all concentrations.

**Table 2:** Light transmission and transparency value at 600 nm of chicken skin gelatin-potato starch film

Sample	Light transmission at different wavelength (%)								Transparency value at 600 nm
	200 nm	280 nm	350 nm	400 nm	500 nm	600 nm	700 nm	800 nm	
**A**	0.01 ± 0.00^a^	0.01 ± 0.00^a^	31.30 ± 4.81^a^	47.85 ± 5.02^a^	57.55 ± 4.031^a^	60.85 ± 3.61^a^	62.85 ± 3.32^a^	64.00 ± 3.00^a^	1.08 ± 0.10^a^
**B**	0.01 ± 0.00^a^	0.01 ± 0.00^a^	31.25 ± 3.75^a^	46.70 ± 3.11^a^	54.85 ± 1.20^ab^	56.25 ± 0.49^b^	58.05 ± 1.06^b^	60.00 ± 0.14^b^	1.30 ± 0.02^a^
**C**	0.01 ± 0.00^a^	0.01 ± 0.00^a^	31.95 ± 3.32^a^	47.05 ± 2.05^a^	55.00 ± 0.42^ab^	57.8 ± 0.14^b^	59.4 ± 0.42^b^	60.35 ± 0.49^b^	1.24 ± 0.01^a^
**D**	0.01 ± 0.00^a^	0.01 ± 0.00^a^	27.95 ± 1.06^a^	44.80 ± 0.28^b^	57.60 ± 1.41^a^	53.15 ± 7.99^c^	64.15 ± 1.48^a^	65.55 ± 1.34^a^	1.18 ± 0.28^a^
**E**	0.01 ± 0.00^a^	0.01 ± 0.00^a^	25.20 ± 1.70^b^	43.50 ± 0.85^b^	49.85 ± 4.74^b^	52.85 ± 6.58^c^	63.35 ± 0.78^a^	64.85 ± 0.92^a^	1.06 ± 0.21^a^
**F**	0.01 ± 0.00^a^	0.01 ± 0.00^a^	24.65 ± 0.07^b^	39.95 ± 2.76^c^	56.00 ± 0.28^a^	60.7 ± 0.71^a^	55.45 ± 5.87^c^	56.35 ± 6.01^c^	0.94 ± 0.04^a^

*A* with 0% of potato starch, *B* with 2% of potato starch, *C* with 4% of potato starch, *D* with 6% of potato starch, *E* with 8% of potato starch, *F* with 10% of potato starch

All data represent mean ± standard deviation; the different superscript letter (^a–b^) in the same column indicate significant difference (*p* *<* 0.05). Values are expressed as mean ± SD (*n* = 3)

With regard to the film characterization against UV light transmission (200–280 nm), the abundant presence of aromatic amino acids in gelatin molecules plays a crucial role in inhibiting UV light transmission. This is because ultraviolet light transmittance across films can lead to inappropriate deterioration of food, thus affecting their nutritional content as well as the overall flavor and texture of food. This is explained by the unique composition of chicken skin gelatin rich with aromatic amino acids content, consisting of 1.22% tyrosine, 1.77% phenylalanine, and 0.04% tryptophan, respectively.^12^ These aromatic amino acids are known to act as sensitive chromophores that aggressively absorb light at wavelengths below 300 nm. In addition to that, it can act as an excellent barrier to UV light, in agreement with a study by Nur Hazirah et al.^24^ Similarly, several studies related to chicken skin gelatin-based film such as,^14,32^ and also,^13^ offered light transmission readings from a low of 0.04 to a high of 0.16 only.

For visible light transmission (350–800 nm), increasing the concentration of potato starch incorporation into chicken skin gelatin-based film significantly lowered the transmission of visible light (*p* < 0.05) for wavelengths 350, 400, 700, and 800 nm. The lower range of data assisted by chicken skin gelatin with potato starch incorporation compared to higher data of control film simply indicates that the incorporation of potato starch did contribute to the light transmission barrier. This might be due to the functionalities imparted by the amylose and amylopectin elements within starch polymers, as they are polarizable and able to oscillate the visible light waves in more than one direction. Hence, this could aid hindering the light transmission by absorbing more incoming light rays.^35^ This is also enhanced by the abundant number of glycosides rings attributed from the starch addition as formed by the less opened film matrix and may hinder the passage of light.^36^ Similar observations can be made for the addition of incorporating materials such as tapioca starch,^14^ CMC,^32^ and also rice flour^13^ into chicken skin gelatin films.

Furthermore, there were no significant differences (*p* < 0.05) for transparency value at 600 nm upon all formulated films, indicating that all of these films assisted a similar degree of transparency at 600 nm. The lower transparency value indicated that the film was more transparent where lower light absorbance was observed.^10^ This was due to the presence of higher potato starch incorporation across the film formulation, which mainly acts as a crosslinking agent in adjusting interfacial interactions in between starch and gelatin polymers, and eventually hinders light absorbance across composite films, thus affecting the transparency of the composite films. Additionally, considering the source of starch used in this study, which is potato starch, excellent transparency properties could possibly be enhanced by the greater number of phosphate monoester groups present within potato starch granules that helps in strengthened intermolecular bonds, thus inducing better light transmission across the film matrix.^22^ Thus, all the films produced in the recent study showed remarkable UV barrier properties. The addition of potato starch into gelatin films enhanced the prevention of UV transmission and thus improved the barrier properties of the blended films against UV light.

### WVP of edible composite films

WVP values for all different formulated chicken skin gelatin-potato starch films, including the control film, are shown in Table 3. From the results, an increasing trend of WVP was observed from films A to D before declining, as shown by films E and F, respectively. WVP of film formulation F was the lowest and showed a significant difference (*p* < 0.05) with formulation D. However, no significant difference (*p* < 0.05) was found for WVP values across film formulations A, B, C, and E, indicating that these films possessed a similar degree of hydrophilicity.

**Table 3:** Water vapor permeability, water solubility, and melting temperature (*T*_m_) chicken skin gelatin-potato starch edible films

Sample	Water Vapor Permeation (x 10^−3^ gmm/m^2^hPa)	Water Solubility (%)	Melting Temperature, T_m_ ( °C)
A	2.10 ± 2.10^ab^	93.66 ± 1.94^a^	45.46 ± 0.49^b^
B	2.26 ± 0.73^ab^	83.61 ± 1.82^b^	44.27 ± 0.78^b^
C	2.44 ± 2.66^ab^	71.01 ± 8.52^b^	45.65 ± 1.69^b^
D	2.66 ± 0.36^a^	87.28 ± 4.66^a^	49.83 ± 1.09^a^
E	2.15 ± 0.20^ab^	76.09 ± 8.79^b^	44.25 ± 1.53^b^
F	2.08 ± 0.29^b^	72.52 ± 4.76^b^	45.61 ± 1.04^b^

*A* with 0% of potato starch, *B* with 2% of potato starch, *C* with 4% of potato starch, *D* with 6% of potato starch, *E* with 8% of potato starch, *F* with 10% of potato starch

All data represent mean ± standard deviation; the different superscript letter (^a–b^) in the same column indicate significant difference (*p* *<* 0.05). Values are expressed as mean ± SD (*n* = 3)

As WVP is strongly dependent on the relative polarity of the polymer used, the presence of water can interact with polymer matrix if the films are strongly hydrophilic, thus leading to increasing values of WVP.^13^ According to Ahmad et al.,^35^ the high levels of additional biopolymers added into blended film matrix were found to lower the free volume availability, thus reducing film compactness. This condition would cause fewer water molecules to be attracted from the environment and produce lower WVP values. The increased WVP values with starch concentration increased may affected the inter and intra molecular structures of blended films. Therefore, the abundance of hydrophilic hydroxyl groups present within amylose concentration of potato starch is favorable to the adsorption and desorption of water molecules. This then allowed greater water affinity and facilitated the migration of water vapor molecules through the film.^37^

These findings agreed with the similar increasing trend of net WVP values reported by Jagadeesh et al.,^30^ which revealed that increasing native potato starch concentrations caused linear increment upon WVP values. Besides, similar increment trends of WVP could be observed with the addition of 0–20% rice flour into chicken skin gelatin-based films.^13^ The findings were also echoed in a study by Nur Hazirah et al.^24^ which reported increased WVP values from 24.40 to 36.38 gmm/m^2^dkPa with increasedxanthan gum incorporation into gelatin-CMC films blended (0–25% xanthan gum incorporation).

### Water solubility of edible composite films

The water solubility of chicken skin gelatin-potato starch edible composite films in various formulations are shown in Table 3. The film solubility of edible chicken skin gelatin films was significantly affected by increased levels of potato starch (*p* < 0.05).

Basically, the effects of the incorporation of potato starch on the solubility of composite films may be associated with inherent hydrophobicity and hydrophilicity of the biopolymers.^38^ This can be observed by the highest value of film A (93.66%) which consisted only of chicken skin gelatin prior to its purpose to act as control film, where great hygroscopic properties of gelatin molecules alone allowed high water affinity, therefore resulting in optimum solubility. On the other hand, the descending trend of water solubility percentage observed might be due to inter and intramolecular interactions between polymer chains in the film matrix.^35^ This was further induced by the hydroxyl groups and amide group of chicken skin gelatin formed during the blending process, leading to strong hydrogen bonds interacting with hydroxyl groups of starch, finally allowing the reduction of available functional groups for water binding molecules.^39^ In addition, the presence of a potato starch as a crosslinker agent also influenced the cohesiveness achieved within the composite biopolymer matrix, thus preventing film solubility in water.^40^

This decline trend also in agreement with studies of chicken skin gelatin-based film with incorporation of several starch sources, for instance 5–25% tapioca starch incorporation (87.91 to 83.29%) by Christine and Sarbon,^14^ and also 15–25% of rice flour incorporation (88.48–82.91%) by Soo and Sarbon.^13^ Furthermore, a higher reading of water solubility detected from chicken skin gelatin-based composite films than other composite films may be due to the higher levels of hydrophilic amino acids (4.66% lysine and 1.22% tyrosine) contained in chicken skin gelatin, which actively allowed complimentary contact with water molecules.^12^

### Thermal properties

The melting transition temperatures or melting points (*T*_m_) of chicken skin gelatin-based films with and without the addition of potato starch are tabulated in Table 3. Thermal properties of chicken skin gelatin-potato starch composite film exhibited a single sharp endothermic peak which associated with only one *T*_m_ values for each of the composite films. Films D possessed significantly different *T*_m_ value against films A, B, C, E, and F, respectively. The highest peak of *T*_m_ exerted by film D with 6% potato starch incorporation.

In this study, increasing *T*_m_ values of chicken skin gelatin-potato starch films were explained throughout the functionalities of potato starch as crosslinker agent in promoting hydrogen bonding interactions and reducing the mobility of biopolymer chains in the film matrix, thus produced heat stable films.^14^ Furthermore, the presence of a crosslinker within the gelatin film matrix proved to improve thermal stability of films by shifting *T*_m_ towards a higher value, as also confirmed by Nur Hazirah et al.^24^ Moreover, the single endothermic peak *T*_m_ obtained from the results indicated that potato starch incorporation into chicken skin gelatin-based film possess good compatibility and thus formed well-homogenous blended film matrix.^39^

These findings were also comparable with other studies of chicken skin gelatin-based films by Soo and Sarbon^13^ and Christine and Sarbon,^14^ in which the same trend increment of data can be spotted even though two endothermic peaks were observed due to the inhomogeneity phenomenon in between gelatin and starch. On the other hand, the *T*_m_ values of control chicken skin gelatin-based film (0% potato starch) from this study are in agreement with the range of *T*_m_ values reported by Nor et al.^7^ They found that the *T*_m_ values ranged from 48.35 °C to 55.51 °C across 0–20% glycerol concentrations. This was comparable with the *T*_m_ values from this study with 30% glycerol used in the development of chicken skin gelatin-potato starch films. In short, film D with the highest *T*_m_ value indicated the homogeneity of continuous composite film matrix achieved by 6% potato starch incorporation into chicken skin gelatin. The findings in this current study have confirmed the compatibility of potato starch within chicken skin gelatin-based film through the occurrence of crosslinking reaction between polymers, indeed optimizing the thermal stability of edible films.

### Film morphology

The scanning electron microscopy images of chicken skin gelatin-potato starch composite films were shown in Table 4. Selective films chosen to be examined were film A, B, D, and F which incorporated with 0, 2, 6, and 10% potato starch, respectively. The purpose of examining these selective films under SEM was to compare the contrast implied by the least, moderate and the most potato starch addition into chicken skin gelatin blended film, which then compared against control film represented by stand-alone chicken skin gelatin film for further validation.

**Table 4:** Scanning electron microscopy images of cross-section surface morphology of chicken skin gelatin-potato starch films at different formulations

Sample	Cross-section	Surface
**A (0% Potato Starch)**		
**B (2% Potato Starch)**		
**D (6% Potato Starch)**		
**F (10% Potato Starch)**		

From microscopy images of the films’ surfaces, it was observed that film A (control) showed a bumpy surface due to some protrusions, as well as slight roughness with wide and noticed crack. This may be due to the discontinuous zones attributed to the only glycerol component presence within single gelatins film, which then lead to preferential channels occurring through drying, and producing noticed cracks along the film network.^41^ However, increasing potato starch incorporated into the chicken skin gelatin films was found to cause reduction of cracks intensity as observed on the surface of films B, D, and F, respectively. Film D showed a smoother, flat and bump-free surface compared to film B because of the ordered structure formed between gelatin and starch polymer chains without inappropriate layering.^35^ This is in agreement with a study by Christine and Sarbon^14^ in which the morphology surface images of gelatin-starch film showed a smoother surface with less protrusions compared to other films. In addition, the smoothness of film surface may be due to inter and intra molecular hydrogen bonding enhanced by the incorporation of starch.^32^ Thus, the incorporation of potato starch within chicken skin gelatin-based blended films has been proven to allow good compatibility between these two polymers thus exerted film with smoother surface.

Focusing on the cross-section microscopic images, it can be confirmed that the presence of cracks was hugely reduced upon increment potato starch concentration into chicken skin gelatin blended film as detailed by films B, D, and F, as compared to the rough obvious long-lined structured cracks shown by film A. The influence of potato starch incorporation into gelatin-based film on film morphology is undeniable as the nature, shape and size of starch granules used as incorporation agent in development of composite film are crucial, as mentioned by De Carvalho and Grosso.^42^ This is shown by the homogeneity of the film matrix between blending gelatin and starch polymer, which then leads to better internal compatibility.^13^ These observations were similar with study by Nazmi et al.,^32^ Christine and Sarbon^14^ and also Soo and Sarbon^13^ in which proven an improvement on smoothness of cross-section morphology via increasing concentrations of CMC, tapioca starch and also rice flour incorporation, respectively. These microscopic results were also found to echo the highest TS value exerted by film D, as if incorporation of potato starch has been proven to improve mechanical properties and performance.

## Discussion

In conclusion, the incorporation of potato starch into chicken skin gelatin-based film was found to influence the mechanical and physical properties of edible films. Potato starch incorporation were improved the viscous-like behavior (*G*″ > *G*′) and also affected the mechanical and physical properties of chicken skin gelatin-potato starch composite films. The TS, Young’s EM, transparency, thermal properties, ultraviolet, microscopy, and visible light barrier transmission increased, and EAB was decreased with increasing potato starch concentrations. However, declining trends were observed on WVP and water solubility of composite films upon increasing potato starch concentration. This may reflect positive prospects on potato starch incorporation as a potential crosslinking agent in order to improve the functionalities of gelatin-based films, especially in the context of food packaging material development.

## Methods

### Materials

Fresh chicken skins were obtained from Jang Maju Enterprise Kuala Terengganu. The skins were chilled in ice during transport to the laboratory. All visible fat was removed mechanically, thoroughly washed and weighed for to determine the wet weight. The cleaned samples were stored at temperature −80 °C for further use. Commercial potato starch was purchased from local supplier in Kuala Terengganu and stored in an airtight container. Glycerol used as a plasticizer was purchased from Mutiara Dinamik Maju Sdn. Bhd. Additional chemicals involved for defatting, extraction and specific analysis, including sodium hydroxide, sulfuric acid, citric acid, and petroleum ether, were obtained from Sigma-Aldrich and were of analytical grade.

### Preparation of chicken skin

Frozen chicken skins were thawed in a cold room (4–5 °C) overnight. The chicken skins were cut into thin 2–3 cm strips before drying overnight in a cabinet drier (FSD-380, Protech, Malaysia) at a temperature of 45 °C. The dried chicken skins were then grounded evenly in a blender (Panasonic, Malaysia) into smaller pieces before being defatted using the Soxhlet method (Soxtec^®^ Avanti System 2055, Foss, Sweden).^43^

### Extraction of chicken skin gelatin

The extraction of chicken skin gelatin was conducted according to Sarbon et al.^12^ Firstly, the defatted dried chicken skins were soaked in sodium hydroxide (0.15%, w/v) before being shaken and stirred slowly for 30 min at room temperature. The solution was then centrifuged (CR 22 N, Hitachi, Japan) at 3500 × *g* for 10 min. The alkaline solution was replaced with a new sodium hydroxide solution every 30 min in order to remove non-collagenous proteins and pigments before rinsing with distilled water. The resulting pellets were then continuously pretreated with sulfuric acid 0.15% (w/v) and citric acid solution 0.7% (w/v), respectively, by performing the same series steps as alkaline pretreatment. Each treatment was repeated three times. The supernatants were removed and the resulting pellets were allowed a final rinse with distilled water. This was followed by centrifugation at 3500 × *g* for 10 min. This continued with overnight extraction in distilled water at 45 °C without stirring. The resultant mixture was filtered out using Buchner funnel with number 4 Whatman filter paper before proceeding to evaporation. The volume of the gelatin solution was reduced to 1/10 by evaporation under vacuum (Rotavapor R25, Buchi, Switzerland). The concentrated gelatin solution was freeze-dried and the resultant dry matter was referred to as ‘gelatin powder’. This gelatin powder was used in combination with various concentrations of potato starch to form film-forming solutions.

### Preparation of edible composite film

Edible composite gelatin FFSs were prepared following Rasid et al.^44^ with certain modifications. Six films formulations were developed with varying concentrations of potato starch based on total weight bases with chicken skin gelatin (4 g) in 100 ml distilled water, as presented in Table 5. Six different blend ratios of chicken skin gelatin to potato starch were prepared: A (100/0), B (100/2), C (100/4), D (100/6), E (100/8), and F (100/10). A total of 30% (w/w) glycerol was added as plasticizer upon all formulations. The complete gelatinization of the potato starch was ensured via dissolution in distilled water and heating with magnetic stirring in a water bath at 90 °C for 30 min. Meanwhile, chicken skin gelatin powder was dissolved in distilled water at 45 °C for 30 min until a clear solution was obtained before being transferred into gelatinized potato starch solution, followed by continuous stirring for another 30 min. This step was followed by the addition of glycerol and then constant stirring for another 30 min until complete dissolution, before being allowed to cool at room temperature. The control film was prepared with the same steps but without any addition of potato starch. The FFSs were cast into flat petri dishes with volumes of 30 g each, followed by drying in a ventilated oven (UNB500, Memmert, Germany) at 45 °C for 3 days. The dried films were carefully peeled off and stored in a desiccator containing silica gel with controlled condition before being subjected for further analysis. Each film formulation was prepared in triplicate.

**Table 5:** Film-forming solution formulation of chicken skin gelatin-potato starch edible composite film

Sample	Chicken skin gelatin (%)	Glycerol (%)	Commercial potato starch (%)	Distilled water (ml)
**A**	100	30	0	100
**B**	100	30	2	100
**C**	100	30	4	100
**D**	100	30	6	100
**E**	100	30	8	100
**F**	100	30	10	100

*A* with 0% of potato starch, *B* with 2% of potato starch, *C* with 4% of potato starch, *D* with 6% of potato starch, *E* with 8% of potato starch, *F* with 10% of potato starch

### Rheological properties of FFS determination

Dynamic viscoelastic properties, through dynamic oscillatory measurement of prepared FFS, were determined according to Rasid et al.^44^ with some modifications. Six different formulation of chicken skin gelatin-potato starch FFS were analyzed for dynamic viscoelasticity using a controlled-stress rheometer (AR2000 RS Advance Rheometer; New Castle, USA) with a cone-plate geometry (cone angle 4°, diameter = 60 mm). Temperature was controlled using a Peltier system. A prepared film-forming solution for each formulation (1 ml) was placed onto the rheometer lower plate’s surface then held for 5 min at room temperature for stress relaxation and temperature equilibration. As each sample was applied to the plate, silicone oil (Sigma cat. no 14615-3) was spread over the outer edge of the sample to prevent evaporation during frequency sweep heating. The extent of the LVR was determined by performing a small-amplitude oscillatory strain (SAOS) sweep test (0.01–20%) with a frequency of 0.1 Hz onto all FFS. The critical strain was defined as the limit strain of LVR which marks the end of the linear stress–strain relation. From the results of these tests, a strain amplitude of 1% within the linear viscoelastic domain in FFS was chosen to perform dynamic oscillatory tests. A dynamic frequency sweep was conducted over a range 0.01–50 rad/s at 25 °C within the identified LVR for each FFS. The results obtained were analyzed using Rheology Advantage Data Analysis V.5.3.1 (TA Instruments). The viscoelastic parameters verified included the storage or elastic (*G*′) modulus and loss or viscous (*G*″) modulus.

## Mechanical properties of edible composite film determination

### Tensile strength, elongation at break (EAB), and Young’s elastic modulus (EM)

The TS, EAB, and EM of chicken skin gelatin-potato starch composite edible film were determined following Nur Hazirah et al.^24^ using a texture analyzer (TA.XT2i Texture Analyzer, Stable MicroSystems, England). The films were cut to 1 × 7 cm and the thickness of each film was measured at five different positions to obtain an average before being attached onto grip pairs of AT/G probe attached to the texture analyzer with 5 kg load cell. The initial gap separation was set to 50 mm. The film strips were then stretched by moving the upper grip at head speed of 1 mm/s until breaking. All three tests were performed in triplicate for each type of film. Values for TS, EAB, and EM were calculated using the following equations:

$${\mathrm{TS}}\,\left( {{\mathrm{MPa}}} \right){\mathrm{ = }}\frac{{{{F}}_{{\mathrm{max}}}\left( {\mathrm{N}} \right)}}{{{{A}}\,\left( {{\mathrm{m}}^{\mathrm{2}}} \right)}},$$

where *F*_max_ is the max load (N) needed to pull the sample apart and *A* is the cross sectional area (m^2^) of the film sample.

$${\mathrm{EAB}}\,\left( {\mathrm{\% }} \right){\mathrm{ = }}\frac{{{{l}}_{{\mathrm{max}}}}}{{l_0}} \times 100,$$

where *l*_max_ is the film elongation (mm) at that moment of rupture and *l*_0_ is the initial grip length (mm) of the sample.

$${\mathrm{EM}}\,\left( {{\mathrm{MPa}}} \right) = \frac{{{\mathrm{Stress}}\,\left( {{\mathrm{MPa}}} \right)}}{{{\mathrm{Strain}}}},$$

where stress is load (N) divided by area (mm^2^) and strain is change in length (mm) divided by original length (mm).

### Film morphology

Film morphology analysis was performed using a scanning electron microscope (SEM), JEOL model JSM-6610LV (Japan) following Soo and Sarbon.^13^ The observations considered the surface and cross sections of film samples, which were first fractured under liquid nitrogen. The prepared samples were mounted on copper stubs perpendicularly to the surface and sputtered with gold coating. This was to ensure that the film was conductive enough to enable the direct flow of 10 kV accelerating voltage at magnification range from ×1500 to 3000.

### Light transmittance and film transparency

The light transmission of gelatin-based films incorporated with potato starch films was measured according to the method by Nur Hazirah et al.,^24^ using a UV–Visible spectrophotometer (UV2601, Rayleigh, China) at wavelengths between 200 and 800 nm. All film samples from each formulation were cut into smaller sizes of 1 cm × 4 cm and placed directly into a test cell. An empty cell was used as a reference, and these tests were conducted in triplicate. The film transparency was calculated using the following equation:

$${\mathrm{Film}}\,{\mathrm{transparency = - }}\frac{{{\mathrm{log}}\,{{T}}_{600}}}{{{x}}},$$

where *T* is the fractional transmittance at 600 nm and *x* is the thickness of film (mm).

### Water vapor permeability (WVP)

The WVP of the edible composite film was measured using a method described by Jahit et al.,^16^ with some modification where circular aluminum cups containing 10 g of silica gel (50% RH) were sealed individually by each film sample with 2 cm × 2 cm length. The thickness of the films was measured using Palmer digital micrometer at six random positions. Each cup was weighed for initial weight, then placed in a desiccator containing distilled water at room temperature. The samples were weighed hourly for 6 h. This test was performed in triplicate for each film. The value of WVP was further calculated using the following equation:

$${\mathrm{WVP = }}\frac{{{{w}}\,\left( {\mathrm{g}} \right){{ \times x}}\,\left( {{\mathrm{mm}}} \right)}}{{{{A}}\,\left( {\mathrm{h}} \right){{ \times t}}\,\left( {{\mathrm{m}}^{\mathrm{2}}} \right){\mathrm{ \times }}\left( {{{P}}_{\mathrm{2}}\, - \,{{P}}_{\mathrm{1}}} \right)\,\left( {{\mathrm{Pa}}} \right)}},$$

where WVP is the water vapor permeability of film (g mm/m^2^ hPa), *w* is the weight gained by the cup (g), *x* is the average film thickness (mm), *A* is the permeation area (m^2^), *t* is time gained (h), and *P*_2_ − *P*_1_ is the difference of partial pressure.

### Water solubility

Water solubility of the edible composite films was determined according to a method by Saberi et al.^45^ with some modifications. Each sample was cut into 15 mm × 40 mm pieces and weighed. The films were then immersed into a 50 ml distilled water in a beaker before being sealed and stirred gently for 24 h at 10 rpm at 25 °C. The remaining undissolved films samples were filtered and dried using a hot air in ventilated oven at 110 °C for at least 2 days until a final constant weight was obtained. This test was performed in triplicate for each type of film. The solubility of the film was calculated according to the following equation:

$${\mathrm{Water}}\,{\mathrm{solubility}}\,\left( {\mathrm{\% }} \right) = \frac{{{\mathrm{initial}}\,{\mathrm{film}}\,{\mathrm{weight-final}}\,{\mathrm{dried}}\,{\mathrm{film}}\,{\mathrm{weight}}}}{{{\mathrm{Initial}}\,{\mathrm{film}}\,{\mathrm{weight}}}} \times 100.$$

### Thermal properties determination by differential scanning calorimetry (DSC)

The thermal properties of edible composite films were performed by using a differential scanning calorimetry (DSC) (Q2000, Modulated TA Instruments, USA) equipped with a cooling device (Intercooler II) as described by Sarbon et al.^46^ About 5 mg of film was weighed using analytical balance (AX224 M-Pact, Sartorius, Germany) and placed in an aluminum sample pan, while another empty pan was used as reference. The pan was then hermetically sealed before heating from 5 to 150 °C at a scanning heating rate of 10 °C/min. Nitrogen gas was used to flush the DSC cell at a flow rate of 20 ml/min to maintain an inert environment. From the melting curve obtained, the melting temperatures (*T*_m_) within the designated area limit were determined, appearing as peaks. These measurements were performed in triplicate.

### Statistical analysis

All analyses were performed in triplicate (*n* = 3) and all collected data were presented by average ± standard deviation evaluated statistically using Minitab Version 14.0 software. One-way analysis of variance (ANOVA) conducted with significant difference of the means was assessed on the basis of Fisher’s test with a confidence level of α < 0.05.

### Reporting summary

Further information on experimental design is available in the Nature Research Reporting Summary linked to this paper.

## Supplementary information

Reporting summary

## Data Availability

Data sharing is not applicable to this article, as no datasets were generated or analyzed during the current study.
